# Influence of the Salmonella Infantis pESI plasmid on disinfectant efficacy when in biofilm

**DOI:** 10.1099/acmi.0.001190.v3

**Published:** 2026-07-27

**Authors:** Emma Davies, Victoria Drauch, Mohammad Alitabar, Darya Fesina, Ludwig Großpointner, Claudia Hess, Francesca Martelli

**Affiliations:** 1 Department of Bacteriology, Animal and Plant Health Agency, Addlestone, KT15 3NB, UK; 2 Clinic for Poultry and Fish Medicine, Department for Farm Animals and Veterinary Public Health, University of Veterinary Medicine Vienna, Veterinärplatz 1, 1210, Vienna, Austria; 3 LVA GmbH, Magdeburggasse 10, 3400 Klosterneuburg, Austria

**Keywords:** antimicrobial resistance, biocide, disinfectant, pESI, plasmid of emerging *Salmonella* Infantis, *Salmonella*

## Abstract

*Salmonella *Infantis strains harbouring multi-drug resistance to high-priority critically important antimicrobials have been isolated globally, giving cause for concern. The serovar is highly persistent throughout the poultry industry and is the fourth most reported serovar linked to zoonotic disease. The serovar’s resistance is attributed to the presence of a megaplasmid termed pESI. Biocides are an important tool to reduce the need to use antimicrobials, including antibiotics. Bacteria can produce a protective exopolysaccharide matrix called biofilm, and biofilm presence can reduce the efficacy of disinfectants. Here, we aimed to assess the role of pESI on disinfectant efficacy and biofilm formation and a contributing factor in serovar persistence. We analysed *S*. Infantis strains isolated within the UK and Austria, using *in vitro* planktonic and biofilm disinfectant efficacy assays. Commercially available, commonly used peroxymonosulphate, chlorocresol and aldehyde–quaternary ammonium compound-based UK poultry disinfectants were assessed. Biofilm formation was evaluated after 72- and 120-h incubation and on a variety of surfaces. Comparative genomic analysis was performed between the UK and Austrian isolates, as well as further globally isolated strains. We identified variation in the presence/absence of antimicrobial resistance genes in both the whole-genome and plasmid sequences, within and between the UK and Austrian strains. Variation in biofilm formation was observed between strains, with greater biofilm formation on non-porous surfaces. Despite this, we could not demonstrate an influence of the pESI plasmid on biofilm formation. Additionally, the presence or absence of the plasmid, or variation observed within the plasmid, did not seem to influence planktonic nor biofilm disinfectant tolerance. Further investigation should be undertaken to identify the influence of pESI on the persistence of *S*. Infantis and to ensure effective prevention and control of the spread of the serovar and the plasmid.

## Data Summary

The authors confirm that all supporting data, code and protocols have been provided within the article or through supplementary data files. All sequence data has been uploaded to public repositories. The Austrian strains were deposited on the NCBI under BioProject PRJNA1381483 (MRS-22-02674: SAMN54106534, MRS-23-00165: SAMN54106535, MRS-23-00825: SAMN54106536 and MRS-23-01610: SAMN54106537). Ten UK strains were previously deposited on EnteroBase[1]. The remaining UK strains were deposited on the NCBI under BioProject PRJNA1403979 (APHA_UK10: SAMN54691484, APHA_UK11: SAMN54691485, APHA_UK12: SAMN54691486, APHA_UK13: SAMN54691487, APHA_UK14: SAMN54691488 and APHA_UK15: SAMN54691489).

## Introduction

The misuse and overuse of antibiotics in human and animal medicine have enhanced the spread of antibiotic resistance. Antibiotic drugs were widely used as treatment for animal disease, as meta-phylaxis (i.e. prophylactic treatment of an entire group of animals regardless of the presence of clinical signs of disease) or as growth promoters (i.e. added to feed to improve on meat quality and volume). Overuse and misuse of antibiotics in these ways are thought to have led to favourable selection of antibiotic-resistant organisms [2]. In recent decades, the use of antibiotics within agriculture and veterinary practices has been restricted within the UK and European Union (EU). Legislation such as the 2019/61 EU regulation on Veterinary Medicines and 2019/4 regulation on Medicated Feed have been enforced to further reduce or stop the use of antibiotics for routine use, prophylaxis and meta-phylaxis. Within human medicine, antibiotics can also be over-prescribed (i.e. used against viral infection) or unsuitably prescribed (such as improper dosages or inappropriate for the target pathogen) [3]. Diagnostic identification of pathogens is slower (i.e. multiple days) than the desired response for treatment, so antibiotics may be prescribed prematurely and potentially inappropriately [3]. Poor infection control, such as hygiene practices and inadequate cleaning, also contributes to the spread of resistant pathogens [4]. Despite greater controls in veterinary and human medicine, the persistence and dissemination of antibiotic-resistant organisms continue to pose a great threat to human, animal and environmental health.

Transfer of antimicrobial-resistant genes (ARGs) between or within bacterial species contributes to the persistence and dissemination of ARGs in the environment [5, 6]. The transfer of ARGs is of great concern, especially when they provide resistance against high-priority, critically important antimicrobials, such as third- and fourth-generation cephalosporins [7]. The increasing presence of multi-drug resistant (MDR) strains is particularly concerning.

The global prevalence of *Salmonella enterica* subspecies *enterica* serovar Infantis has increased drastically in recent decades, largely due to the emergence of antimicrobial-resistant (AMR) strains [8–16] that have disseminated clonally. *S*. Infantis is the fourth most reported serovar in the EU, accounting for human gastrointestinal infection (salmonellosis) [17]. Furthermore, it is the most common serovar found in broiler and broiler products [12, 17] and makes up the largest proportion of MDR *Salmonella* isolated from poultry (74.4% for broilers, 32.4% for laying hens and 31.5% in turkeys) [18]. *S*. Infantis has been identified globally to harbour various ARGs, including those conferring resistance against critically or highly important antibiotics [7], such as gentamicin [14, 19].

A comparative study in 2014 [11] identified the presence of an MDR megaplasmid in emerging populations of *S*. Infantis, the plasmid of emerging *Salmonella* Infantis (pESI). The plasmid was found to harbour multiple ARGs, encoding resistance to at least trimethoprim, tetracycline and sulphonamides. Since then, pESI and pESI-like *S*. Infantis have been identified globally [8–10, 12–14, 19–30]. The plasmid can also harbour a region of high virulence-associated genes (yersiniabactin operon) as well as resistance to beta-lactams and mercury [11, 12]. These virulence genes may be involved in adhesion, colonization and proliferation, some of which have previously been linked to biofilm formation [11]. Additionally, the plasmid has been found to carry the quaternary ammonium compound (QAC) resistance gene (qacEΔ1) [11], suggesting potential resistance to disinfectants [11].

Disinfection biosecurity measures are heavily relied upon to prevent, respond to and control microbial contamination. However, co-resistance between antibiotics and disinfectants is increasingly observed and often poorly understood [31]. Disinfectants target pathogens in a variety of ways such as acting against cell membranes, inner cell components, oxidation and enzyme inhibition [32]. The mechanisms used by pathogens to protect themselves against disinfectants are like those observed against antibiotics. These may include enzyme inactivation, efflux pumps, formation of biofilm, target modification and changes in the cell surface [33, 34]. Biofilms occur when bacteria congregate and adhere to surfaces and form a protective extracellular polysaccharide matrix. The removal of biofilms is a major issue across agricultural, food production and veterinary sectors [35, 36]. Commercial disinfectants are typically proven effective against planktonic bacteria or bacteria dried onto surfaces, as opposed to in the biofilm state [37]. Higher concentrations of disinfectant, as well as physical removal from surfaces, are required for effective control of biofilm [38–42]. The efficacy of disinfectants, both in the planktonic and biofilm state, has also been found to be strain specific in *S*. Infantis [43]. Consequently, this may explain the persistence and rapid global dissemination of MDR *S*. Infantis.

Understanding the role of the pESI plasmid on tolerance to disinfectants and its role in biofilm formation is critical in ensuring effective prevention and control measures for human, animal and environmental health. Furthermore, understanding the possible global variations in pESI and pESI-like disinfectant resistance is of utmost importance and needs to be assessed. Therefore, this study aimed to compare biofilm formation and disinfectant tolerance of *S*. Infantis harbouring pESI and pESI-like plasmids isolated from Austria and the UK.

## Methods

### Isolate selection

**Fig. 1.**
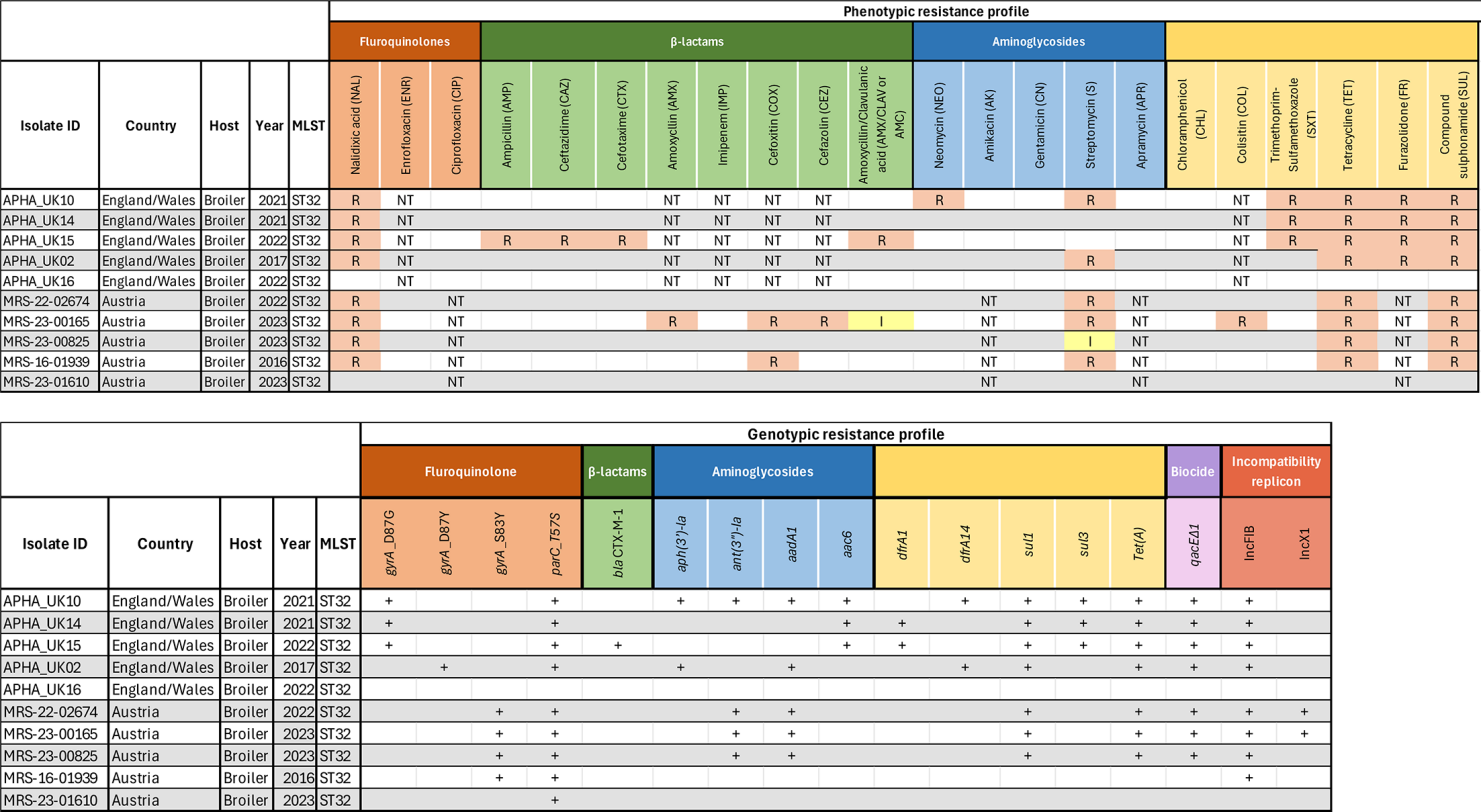
Ten *S*. Infantis, isolated from the UK and Austria. The country, host and year of isolation are included, alongside the MLST. Both phenotypic (top) and genotypic (bottom) resistance profiles are shown. In the UK, the Kirby–Bauer disc diffusion method was used to screen for antibiotic susceptibility against a panel of 16 antibiotics [nalidixic acid (NA), tetracycline (TET), neomycin (NEO), ampicillin (AMP), furazolidone (FR), ceftazidime (CAZ/TAZ), sulphamethoxazole–trimethoprim (SXT), chloramphenicol (CHL), amikacin (AK), amoxicillin/clavulanic acid (AMC), gentamicin (CN), streptomycin (S), compound sulphonamides (SUL), cefotaxime (CTX), apramycin (APR) and ciprofloxacin (CIP)]. In Austria, isolates were screened via broth microdilution against a panel of 19 antibiotics [AMX/CLAV, AMP, amoxicillin (AMX), cefazolin (CEZ), cefoxitin (COX), CTX, CAZ, CHL, COL, NA, enrofloxacin (ENR), CN, S, NEO, imipenem (IMP), trimethoprim (TRI), TET, SXT and SUL]. UK susceptibility was assessed using the British Society for Antimicrobial Chemotherapy (BSAC) cut-offs [44]. Austrian susceptibility was assessed using CLSI VET (VET01S) cut-offs. Isolates were sequenced using Illumina MiSeq System (Illumina Inc, CA, USA). Unicycler [45] was used to assemble, and AMRFinderPlus [47] and staramr v.0.9.1 [48] were used to identify antibiotic, disinfectant and virulence genes. NT, not screened against; R, resistant; I, intermediate; +, gene present conferring resistance.

Ten *S*. Infantis isolates – five each from Vienna and the UK – were chosen for biofilm and disinfectant screening. Prior antibiotic susceptibility tests guided the selection of potential pESI/pESI-like strains, which were confirmed via whole-genome sequencing (WGS). Details of isolate selection are provided below; see Fig. 1 for the selected panel of strains.

To enable selection of five UK-isolated strains, a larger panel was first screened. Fifteen isolates – nine from published work [1] and six from ongoing research – were obtained from the Animal and Plant Health Agency (APHA) culture collection archive. These were collected from broiler poultry between 2011 and 2022. Antibiotic susceptibility was assessed using the Kirby–Bauer disc diffusion method, with suspected pESI/pESI-like strains showing resistance to tetracycline, sulphonamides and trimethoprim–sulphamethoxazole, per BSAC guidelines [44]. See Fig. 1 for the antibiotic panel. All strains underwent WGS using the Illumina MiSeq System (Illumina Inc, CA, USA). Sequences were assembled with Unicycler [45]. Quality Assessment Tool (QUAST) [46] was used to evaluate the quality of the assemblies (acceptable parameters: N50 >50,000, contigs <200 and base pairs between 4.5 million and 5.2 million) (Material S3, available in the online Supplementary Material). Antibiotic and disinfectant resistance and virulence genes were identified using AMRFinderPlus [47] and staramr [48]. Based on AMR profiles, ten groups emerged (Material S1); based on relevance to human and animal health and similarity or divergence from global pESI/pESI-like plasmids, one isolate from four of the AMR profiles was selected.

Austrian isolates, sourced from broiler environmental samples (2016–2023) at the University of Veterinary Medicine Vienna, were selected from an ongoing *S*. Infantis project. AMR profiles were assessed using broth microdilution (Micronaut, Merlin Diagnostika, Bornheim-Hersel, Germany), covering 19 antimicrobials across 10 classes (Fig. 1). Both MDR and non-MDR strains were included.

### Comparative WGS analysis

WGS enabled comparison of Austrian and UK *S*. Infantis isolates, alongside global pESI/pESI-like strains (Material S2), focusing on antimicrobial, virulence and disinfectant genes. Plasmid sequences from eight isolates were assembled using MOB_recon [MOB-suite (https://github.com/phac-nml/mob-suite)], annotated with Bakta [49] and visualized via BRIGG [50] using the Israeli pESI_119944 as reference. Phylogenetic analysis of the ten UK and Austrian core genomes was conducted using SNIPPY (https://github.com/tseemann/snippy) and iqtree2 (https://github.com/iqtree/iqtree2) and visualized using iTOL.embl. SNP distances were assessed by snp-dists (https://github.com/tseemann/snp-dists); isolates differing by fewer than five SNPs were considered epidemiologically linked [51].

### Disinfectant selection and preparation

Three UK commercial poultry disinfectants, which are commonly applied on farms in the UK, were chosen in consultation with the poultry industry. Table 1 lists the products by chemical class and details the concentrations and methods used. Disinfectants were prepared fresh for each test in the World Health Organization (WHO) hard water [calcium chloride 2H_2_O: 0.30 g (Chemlab, CL00.0303.100), magnesium chloride 6H_2_O: 0.14 g (VWR, 25108.260) and purified water (Merck, 307297)].

**Table 1.** Commonly used UK commercial poultry disinfectant products selected for preliminary disinfectant efficacy testing The chosen concentrations (range and single dilution) prepared for each test method are listed.

Disinfectant class	Chemical component	MIC concentration range	Coupon assay concentration
Peroxymonosulphate	Pentapotassium bis(peroxymonosulphate) bis(sulphate); benzenesulfonic acid, C10-13-alkyl derivs, sodium salts; malic acid; sulphamidic acid; potassium hydrogen sulphate; dipotassium disulphate; sodium toluene sulphonate; dipentene	1:2.5–1:640	1:160
Aldehyde–QAC	QACs, benzyl-C12-16-alkyldimethyl, chlorides; didecyldimethylammonium chloride; glutaraldehyde; propan-2-ol	1:33–1:16,896	1:528
Chlorocresol	propionic acid, chlorocresol, sulphonic acid	1:50–1:3,200	1:150

### Bacterial recovery

Austrian isolates were stored in Brain Heart Infusion (CM1135, Oxoid, Wesel, Germany) mixed with 20% glycerol (Herba Chemosan Apotheker-AG, Vienna, Austria) stocks at −20 °C and recovered on blood agar [Columbia sheep blood agar (COS), bioMérieux, Marcy-l'Etoile, France]. UK isolates were stored on cryovial beads (Pro-Lab Diagnostics, Wirral, UK) at −80 °C and recovered on 5% sheep blood agar (SBA; Oxoid Limited, Altrincham, UK). All were incubated at 37±2 °C for 24 h and freshly recovered for each experiment.

### Crystal violet microtiter plate assay

After recovery, as per 5.4 bacterial recovery, 1–2 colonies were cultured in Luria–Bertani (LB) broth (no salt) (VWR, 1.10285.0500) and statically incubated at 37±2 °C for 24 h in biological triplicate. Overnight cultures were serially diluted and plated to establish an inoculum density of between 6×10^9^ to 9×10^10^ c.f.u. ml^−1^. The overnight cultures were diluted (20 µl in 180 µl) in technical triplicate in an uncoated polypropylene 96-well plate (Nunc Nunclon Delta Multiwell Plate F96, Cat. No. 167008, Scientific Laboratory Supplies Limited). At least one triplicate set of wells containing 200 µl blank broth was included. Plates were incubated statically without a plate seal at 20±2 °C for 72 and 120 h, after which excess broth was removed by pipetting and wells were washed thrice with 300 µl sterile distilled water. Wells were stained with 2% crystal violet stain (Merck, Cat. No. HT901-8FOZ) for 15 min, the stain was removed and the wells were washed thrice, before resolubilizing the stain with 95% ethanol for at least 10 min. Absorbance at 595 nm was measured using a Tecan™ plate reader (Infinite® M Nano, Tecan Austria GmbH, Grödig, Austria). This was performed in biological triplicate, thus resulting in a total of nine values per strain (i.e. biological triplicate and technical triplicate). From each biological replicate, the three blank wells were averaged and subtracted from each of the corresponding technical replicates, giving three values which were then averaged to give the mean OD_595_ for each biological replicate (i.e. three values per strain). These three mean values were averaged to give the mean OD_595_ for each strain. The mean OD_595_ values were used to calculate biofilm formation (see Table 2), classified as previously described [52].

**Table 2.** Quantification of biofilm formation as per Stepanović *et al*. [52]

Biofilm formation	Calculation
Strong producer	Mean OD_595_>4xOD_b_
Moderate producer	2xOD_b_<mean OD_595_≤4xOD_b_
Weak producer	OD_b_<mean OD_595_≤2xOD_b_
Non-producer	Mean OD_595_≤OD_b_

OD_b_, mean OD_595_ of nine blank control wells across three biological replicates.

### MIC and minimum bactericidal concentration

Disinfectant MIC testing was performed in technical and biological triplicate. The disinfectant MIC plates were prepared in-house. The disinfectants were prepared to 2× the desired highest concentration (Table 1), to account for dilution within the plate upon inoculation with bacteria, used within 2 h of preparation. The selected concentration was that of the Department for Environment, Food and Rural Affairs (DEFRA) Disinfectant Approvals General Orders concentration (approved in the UK for use in the event of an outbreak of notifiable disease and recommended for general use) and was adjusted as required (i.e. for the peroxymonosulphate product, stronger concentrations were included throughout the duration of the study). Doubling serial dilutions were performed in the WHO hard water within the 96-well plate, with one row as a WHO hard water growth control.

Bacterial inoculum was standardized to 0.5 McFarland, prepared using a densitometer in 5 ml sterile water (YT3339, Thermo Fisher Diagnostics Ltd, Hemel Hempstead, UK) and briefly vortexed. Ten microlitres was inoculated into Mueller–Hinton broth (YT3462, Thermo Fisher Diagnostics Ltd), and the tube cap was replaced with a Sensititre™ dosing head before inoculation of 50 µl using the Sensititre™ auto inoculator. The plate was incubated at 37±2 °C for 24 h and then read with a Vizion™ plate reader. MIC was determined as the lowest concentration with no visible growth.

Minimum bactericidal concentration (MBC) was determined using both solid and liquid recovery methods. Ten microlitres from the MIC, 2xMIC and 4xMIC wells were plated on 5% SBA. To assess MBC at the same time point within both solid and liquid recovery methods, a neutralizer:resuscitation broth was added 1:1 in the 96-well plate. QAC products used lecithin-based neutralizer (Tween 80, SLS, Cat. No. CHE3854; saponin, Sigma, Cat. No. 47036-250f; lecithin, VWR, Cat. No. 24966.180), whilst all other classes used nutrient broth No. 2 [Oxoid Nutrient Broth No. 2 (dehydrated), Thermo Fisher Scientific, Cat. No. CM0067b) + horse serum (Gibco™ Horse Serum, heat inactivated, New Zealand origin, Fisher Scientific, Cat. No. 10368902). Nutrient broth No. 2 was used as the resuscitation broth. The 96-well plates were incubated for 72 h at 37±2 °C, and the 5% SBA plates were incubated for 24 h at 37±2 °C, before observing for the presence or absence of turbidity and growth, respectively. MBC was established as the lowest concentration with no visible growth. The final MBC was taken as the lowest concentration between the methods to produce no visible growth.

### Surface coupon assay

Biofilm disinfectant tolerance was assessed on concrete [Blue Circle Mastercrete quick set cement and sharp sand, prepared in-house in a silicone mould as per manufacturer’s instructions (10 mm^3^)] and polyvinyl chloride (PVC) (satin finish, 10×20×1.5 mm, Hygienic Plastics Supplies Ltd., Lancashire, UK). Concrete coupons were cured at room temperature for a week and then removed from the mould before curing for a further week. The coupons were washed in a dishwasher without detergent at 60 °C for two 60-min cycles to counter alkaline properties. Both coupon types were sterilized in 95% ethanol solution for at least an hour, before repeating and drying under sterile conditions. Glass beads (5 mm soda lime glass beads (Z265942; Sigma-Aldrich, MO, USA), used for biofilm removal, were autoclaved sterilized in an autoclave at 121 °C for 15 min. When required, the beads were decanted into sterile glass vials (Sample vials, VWR, 216-3082) using sterile tweezers.

After recovery, as per 5.4 bacterial recovery, 2–3 bacterial colonies were inoculated into 10 ml LB broth (to achieve ~1×10^7^ c.f.u. ml^−1^). Then, 1.5 ml of each bacterial suspension was aliquoted into 6 wells of a 12-well plate (Nunc flat-bottom 12-well clear polystyrene plates with the Nunclon™ Delta surface treatment and lid; Scientific Laboratory Supplies Ltd). Using sterile tweezers, three of each coupon type were placed into the 12-well plate, long edge into the broth and half exposed to the air. Plates were incubated for 72 h at 20±2 °C. Bacterial inocula were serially diluted in PBS and plated onto LB agar [Carl Roth, LB Agar (Luria/Miller), Art.-Nr.X969.1], incubated at 37±2 °C for 24 h, before plate counts were performed.

After incubation, the coupons were individually placed into glass vials using sterile tweezers. Loosely adhered bacteria were removed via gentle washing by pipetting 9 ml sterile saline (0.85% physiological saline, Carl Roth, sodium chloride, Art.-Nr.HN00.2), repeated thrice. Disinfectant concentrations were prepared as described in Table 1. For each isolate and coupon type, 5 ml disinfectant was added to two coupons and incubated at 20±2 °C for 30 min. The WHO hard water was added to the third coupon to act as an enumeration control. After the contact time, the disinfectant was removed, and 5 ml Dey–Engley neutralizer (D3435, Sigma-Aldrich, MO, USA) was added and incubated at room temperature for at least 5 min. The neutralizer was then removed, and 5 ml sterile saline and 15 sterile glass beads were added. The vials were then vortexed for 1 min at the lowest speed, before 1:10 dilutions in PBS were prepared. Then, 100 µl of each dilution was spread plated onto LB agar and incubated at 37±2 °C for 24 h, before plate counts were performed. Tests were performed in biological triplicate.

### Graphical and statistical analysis

R Core Team [version 4.5.2 (2025-10-31)] [53] and R Studio™ (version 2025.05.1) [54] were used to perform statistical analyses and graphical presentation (ggplot2 [55]). For the crystal violet microtiter plate assay (CVA), the biological replicate OD_595_ (*n*=3), calculated as previously described, was used to assess significant differences between the ten isolates for each incubation duration using the Kruskal–Wallis test followed by Dunn’s post hoc test (Bonferroni correction, *P≤*0.05).

For the surface coupon assay, log bacteria/biofilm was calculated following enumeration for each surface and strain. Per biological replicate, the log bacteria/biofilms of the two technical replicates were averaged (*n*=3). The log bacteria/biofilm of the single WHO hard water control technical replicate was then used to calculate the log reduction/biofilm; this was repeated for each biological replicate (*n*=3); this was used within statistical analysis. The average log reduction/biofilm of the three biological replicates was used for graphical presentation, with error bars depicting the sd between the log reduction/biofilm of the three biological replicates. Average log bacteria/biofilm for the WHO hard water control was calculated using the log bacteria/biofilm obtained across all 11 tests for each isolate. Normality was evaluated using Shapiro–Wilk tests. Significant differences in biofilm formation for the same isolate between the different surfaces were assessed using a paired t-test (*P≤*0.05). Significant differences between all ten isolates on each separate surface were analysed with either Kruskal–Wallis or ANOVA (*P≤*0.05). Analysis was followed by Dunn’s post hoc test (Bonferroni correction, *P≤*0.05).

## Results

### Isolate selection, WGS and bioinformatic analysis

Fifteen UK *S*. Infantis strains were screened for antibiotic, disinfectant and virulence genes and grouped into ten AMR profiles (Material S1). All carried the pESI IncFIB(pN55391) replicon; two also harboured IncFII(29) (APHA_UK06) and IncX4 (APHA_UK09). All strains had the parC(T57S) SNP conferring fluoroquinolone resistance and one of the three gyrA SNPs conferring nalidixic acid resistance [*gyrA*(D87G), *gyrA*(D87Y) and *gyrA*(S83Y)]. Only one isolate (APHA_UK05) was non-MDR, only harbouring resistance to fluoroquinolones; the rest were multi-drug-resistant.

Five isolates – four harbouring pESI/pESI-like plasmids and one pan-susceptible – were selected for screening of biofilm formation and disinfectant tolerance. Two isolates (APHA_UK02 and APHA_UK10) were chosen which harboured pESI plasmids conferring aminoglycoside resistance [*ant(3″)-la*, *ant(3″)-la* and *aph(3′)-la*, respectively]. APHA_UK10 also differed from APHA_UK02 by the SNP conferring resistance to nalidixic acid [*gyrA*(D87H) as opposed to *gyrA*(D87Y)]. Another isolate (APHA_UK15) was selected harbouring a pESI-like plasmid conferring resistance to beta-lactams (*bla*_CTX-M-1_). A fourth isolate (APHA_UK14) was chosen for its similarity to APHA_UK15, other than for the absence of the beta-lactam resistance gene. A fully antibiotic-susceptible *S*. Infantis (APHA_UK16) was also selected to complete the UK panel (Fig. 1).

Eight *S*. Infantis strains (four UK and four Austrian) with pESI/pESI-like plasmids were included in this study; all carried antibiotic, disinfectant and virulence genes and characteristic pESI incompatibility replicons such as *dfrA1, dfrA14*, *sul1*, *sul3*, *tet(A)*, *qacE∆1* and IncFIB(pN55391). Some also harboured genes conferring resistance to beta-lactams and or aminoglycosides. All ten strains harboured at least one chromosomal fluoroquinolone resistance gene [*gyrA*(D87G), *gyrA*(D87Y), *gyrA*(S83Y) and/or *parC*(T57S)]. All five Austrian isolates, including the otherwise antibiotic-susceptible isolate (MRS-23-01610), harboured the same two genes: *gyrA*(S83Y) and *parC*(T57S). All four UK pESI isolates harboured *parC*(T57S), three of which harboured the nalidixic acid *gyrA*(D87G) resistance gene, whilst one harboured an alternative mutation [*gyrA*(D87Y)].

**Fig. 2.**
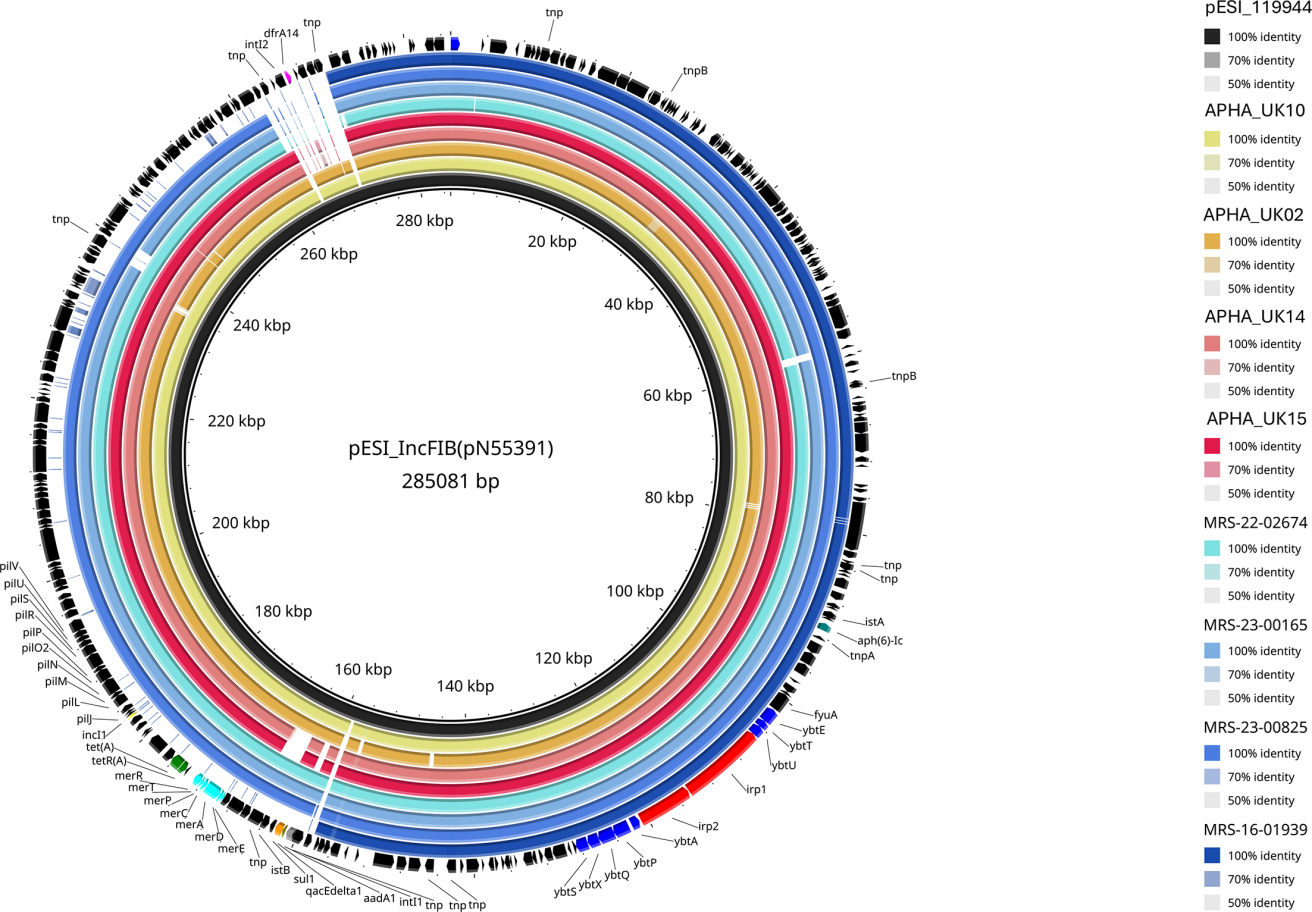
Alignment and annotation of the Israeli-isolated pESI_119944 [11] strain with the four UK and four Austrian pESI/pESI-like strains. MOB_recon (https://github.com/phac-nml/mob-suite) was used to generate plasmid sequences from the short-read sequence assemblies; an incomplete plasmid sequence was generated for MRS-16-01939. Bakta [49] was used for annotation and BRIGG [50] for visualization.

Alignment of the eight plasmid sequences, using blast and BRIGG (Fig. 2), showed high similarity with the Israeli pESI_119944 (query cover >99%, percentage identity >99.91%). Like pESI_119944, APHA_UK10 and APHA_UK02 both harboured *dfrA14* in one region and harboured *sul1*, *aadA1* and *tet(A*) within another. Three Austrian isolates (MRS-22-02674, MRS-23-00165 and MRS-23-00825) also harboured the *aadA1*, *sul1* and *tet(A*) genes within the same region, though lacked any *dfrA* gene. Within this region, APHA_UK15 lacked *aadA1* but harboured *bla*_CTX-M-1_, whereas APHA_UK14 lacked both *aadA1* and *bla*_CTX-M-1_. Both strains harboured *dfrA1* in this second region. Seven strains harboured the disinfectant gene *qacE∆1*. All eight harboured the IncFIB(pN55391) replicon. Two Austrian isolates harboured an additional replicon, IncX1. A much shorter plasmid sequence was obtained following use of Mob_recon for MRS-16-01939; this strain harboured the IncFIB(pN55391) replicon in the absence of any antibiotic resistance genes.

**Fig. 3.**
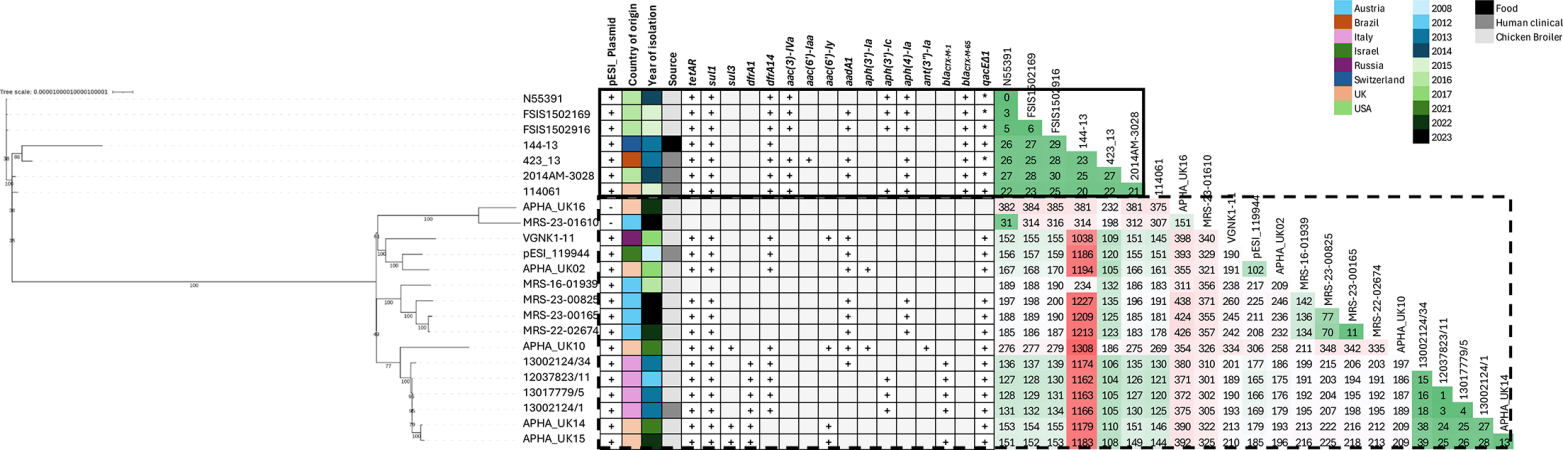
Core genome phylogenetic tree containing 13 globally isolated published pESI/pESI-like *S*. Infantis isolates alongside this study’s five UK and five Austrian pESI/pESI-like isolates. The Israeli pESI_119944 strain was used as the reference. The table depicts the presence of the plasmid, country of origin, year of isolation and source of isolation, as well as the presence of key antibiotic resistance genes (indicated by +). The heat map matrix annotates the number of SNPs between each isolate. A predominantly (North/South) American clade can be identified (solid black box), as well as a European clade (dashed black box). Phylogenetic analysis tools included SNIPPY (https://github.com/tseemann/snippy), iqtree2 (https://github.com/iqtree/iqtree2), iTOL.embl and snp-dists (https://github.com/tseemann/snp-dists). Isolates differing by <5 SNPs are epidemiologically linked [51].

Following whole-genome phylogenetic analysis (Fig. 3), two distinct clades were identified: clustering by the presence or absence of the *bla*_CTC-M-65_ gene. Strains harbouring *bla*_CTX-M-65_ were predominantly isolated from North or South America or were isolated from food or human samples. The other clade consisted of European or Middle Eastern strains; the ten UK and Austrian isolates were located within this clade. The two non-pESI isolates (APHA_UK16 and MRS-23-01610) were more closely linked to one another (151 SNPs) than the pESI/pESI-like strains. They differed from the Russian, Israeli and one of the UK isolates (APHA_UK02) by between 321 and 398 SNPs. APHA_UK02 was most like the Israeli pESI_119944 (102 SNPs). The four Austrian pESI/pESI-like strains clustered together, ranging by between 11 and 142 SNPs. The UK strain APHA_UK10 differed by 306 SNPs from APHA_UK02 and between 211 and 335 SNPs from the four Austrian strains; it was more closely related to APHA_UK14 and APHA_UK15, differing by 209 SNPs. These latter two strains, differing by only the presence/absence of *bla*_CTX-M-1_, were closely related, differing by only 13 SNPs.

### Crystal violet microtiter plate assay

**Fig. 4.**
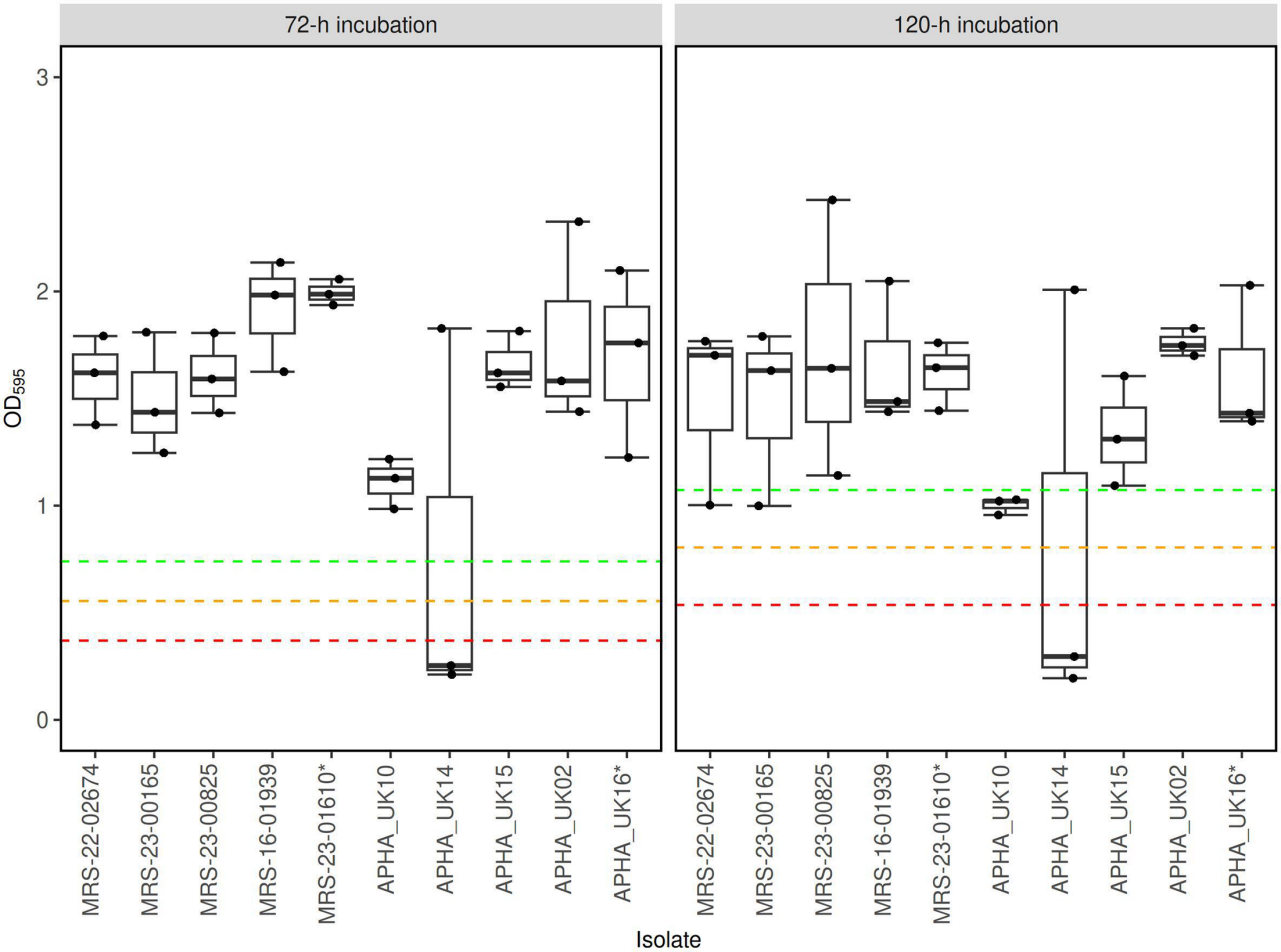
Box and whisker plots showing biofilm formation of ten isolates (five UK and five Austrian) established via the CVA following 72- and 120-h incubation at 20±2 °C. The OD_595_ of three technical and three biological replicates (*n*=nine) are depicted. The dotted lines plot the minimum OD_595_ required for classification as a weak (red), moderate (orange) and strong (green) biofilm former, according to the classification by Stepanović *et al*. [51, 52]. The asterisk (*) depicts isolates without the pESI/pESI-like plasmid.

All ten strains produced biofilm following both 72- and 120-h incubation at 20±2 °C (Fig. 4). Biofilm formation of the antibiotic-susceptible isolates (APHA_UK16 and MRS-23-01610) did not differ considerably from the eight pESI isolates.

No significant differences (*P≤*0.05) were found between the strains under both incubation conditions (72- and 120-h incubation). Despite this, Fig. 4 shows variation between strains; APHA_UK14 was the poorest biofilm former, and more variation was observed between the UK isolated strains than between the Austrian isolated strains.

Biofilm formation was similar between the incubation durations, with marginally higher average OD_595_ (in eight out of ten isolates) following 72-h incubation (Table 3). A larger range in average OD_595_ was observed following 72-h incubation than following 120 h (1.23 range OD_595_ for 72 h and 0.93 range OD_595_ for 120-h incubation). The average OD_595_ range varied amongst UK and Austrian isolates, respectively, with more variation observed amongst UK isolates (up to 1.03 OD_595_) than Austrian isolates (up to 0.51 OD_595_) (Table 3).

**Table 3.** Average OD_595_ (*n*=9) of ten *S.* Infantis strains Three biological and technical replicates were performed. Biofilm classification, as per Stepanović *et al*. [52], is stated following 72- and 120-h incubation at 20±2 °C. Green indicates a strong biofilm former, whilst orange indicates a moderate biofilm former.

Isolate ID	Duration	Average OD_595_	Biofilm classification	Duration	Average OD_595_	Biofilm classification
MRS-22-02674	72	1.60	Strong	120	1.49	Strong
MRS-23-00165	72	1.50	Strong	120	1.47	Strong
MRS-23-00825	72	1.61	Strong	120	1.74	Strong
MRS-16-01939	72	1.91	Strong	120	1.66	Strong
MRS-23-01610	72	1.99	Strong	120	1.62	Strong
APHA_UK10	72	1.11	Strong	120	1.00	Moderate
APHA_UK14	72	0.76	Strong	120	0.83	Moderate
APHA_UK15	72	1.66	Strong	120	1.34	Strong
APHA_UK02	72	1.78	Strong	120	1.76	Strong
APHA_UK16	72	1.69	Strong	120	1.62	Strong

### MIC and MBC

**Fig. 5.**
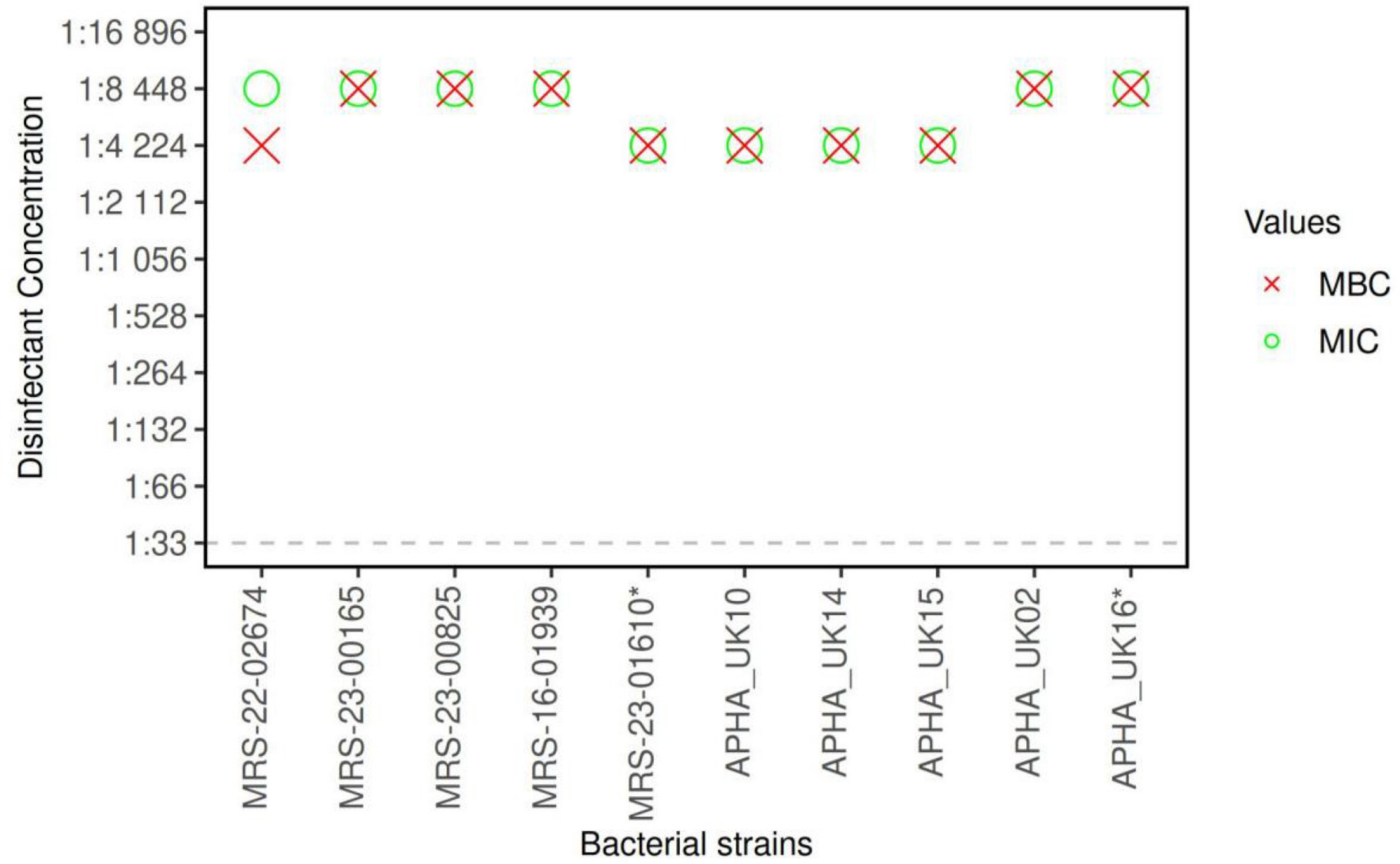
The MIC (green circle) and MBC (red cross) for the aldehyde–QAC product against the ten isolates. Mode technical and biological triplicate (nine replicates). The asterisk (*) depicts isolates without the pESI/pESI-like plasmid.

**Fig. 6.**
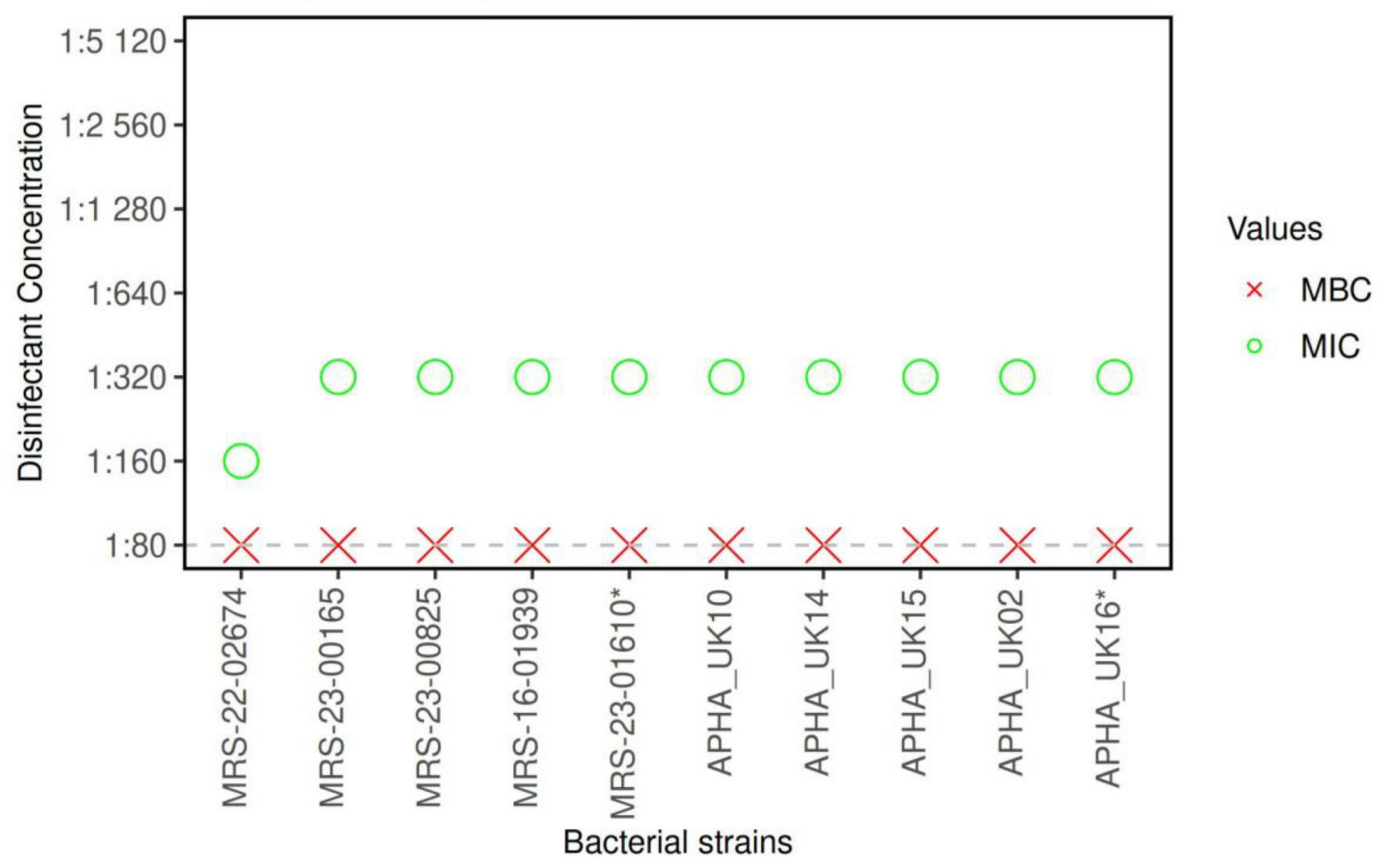
The MIC (green circle) and MBC (red cross) for the peroxymonosulphate product against the ten isolates. Mode technical and biological triplicate (nine replicates). The asterisk (*) depicts isolates without the pESI/pESI-like plasmid.

**Fig. 7.**
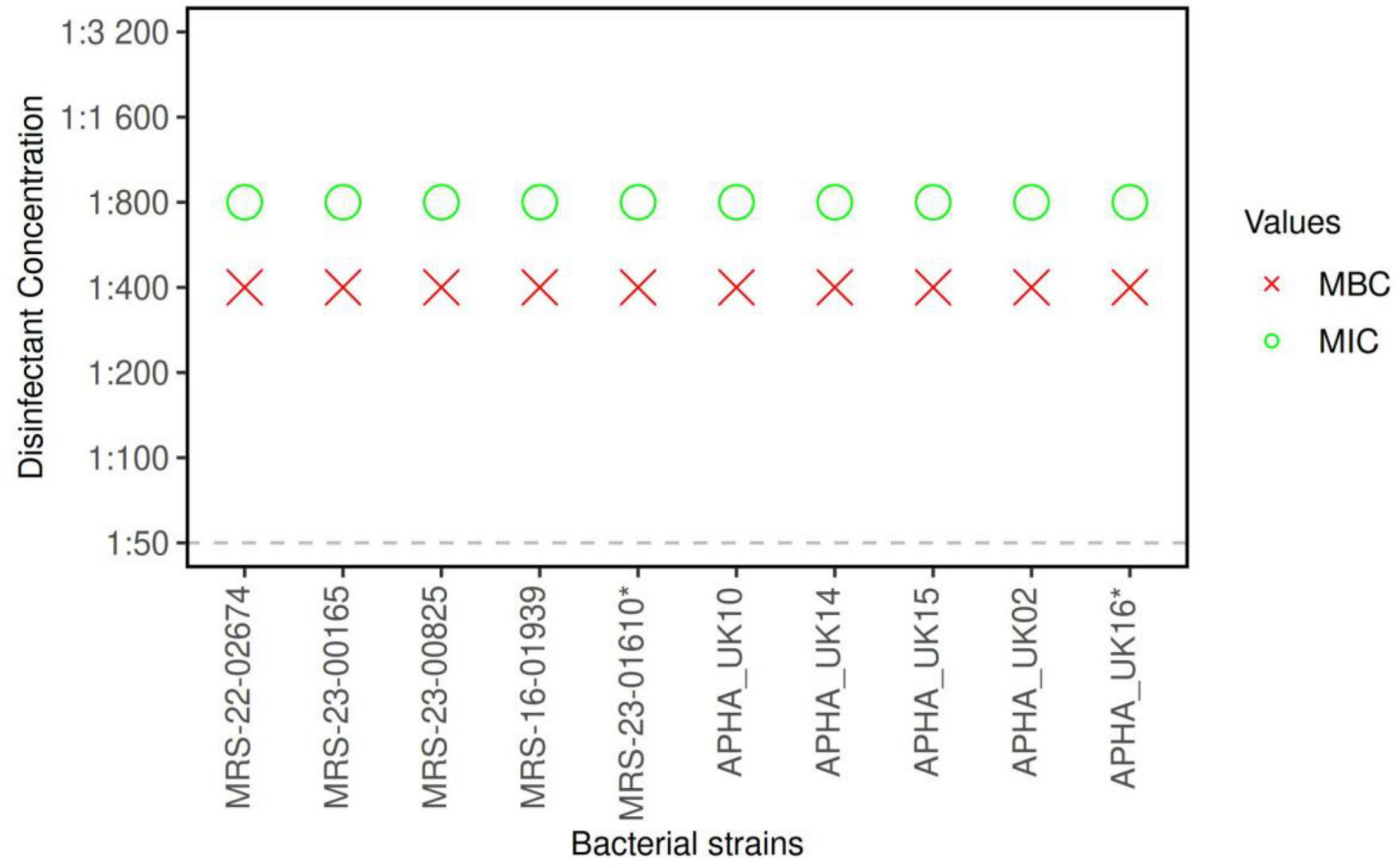
The MIC (green circle) and MBC (red cross) for the chlorocresol product against the ten isolates. Mode technical and biological triplicate (nine replicates). The asterisk (*) depicts isolates without the pESI/pESI-like plasmid.

MIC and MBC varied between the isolates and disinfectant products. All ten isolates were most susceptible (MIC) to the aldehyde–QAC product [up to eightfold weaker concentrations than the planktonic recommended concentration (GO)] (Fig. 5). All ten isolates were most tolerant to the peroxymonosulphate product (Fig. 6), requiring up to only twofold weaker concentration than recommended against planktonic bacteria. Across all three products, the ten isolates varied in MIC by up to twofold. MICs remained the same for the chlorocresol (Fig. 7) product, and only one isolate (MRS-22-02674) required a higher concentration for the peroxymonosulphate product (Fig. 6).

MBC was the same for all ten isolates for both the chlorocresol and peroxymonosulphate products, whereas MBC varied by up to onefold for the aldehyde–QAC product. MBC was the same as the MIC for the aldehyde–QAC product for nine isolates.

### Surface coupon assay

**Fig. 8.**
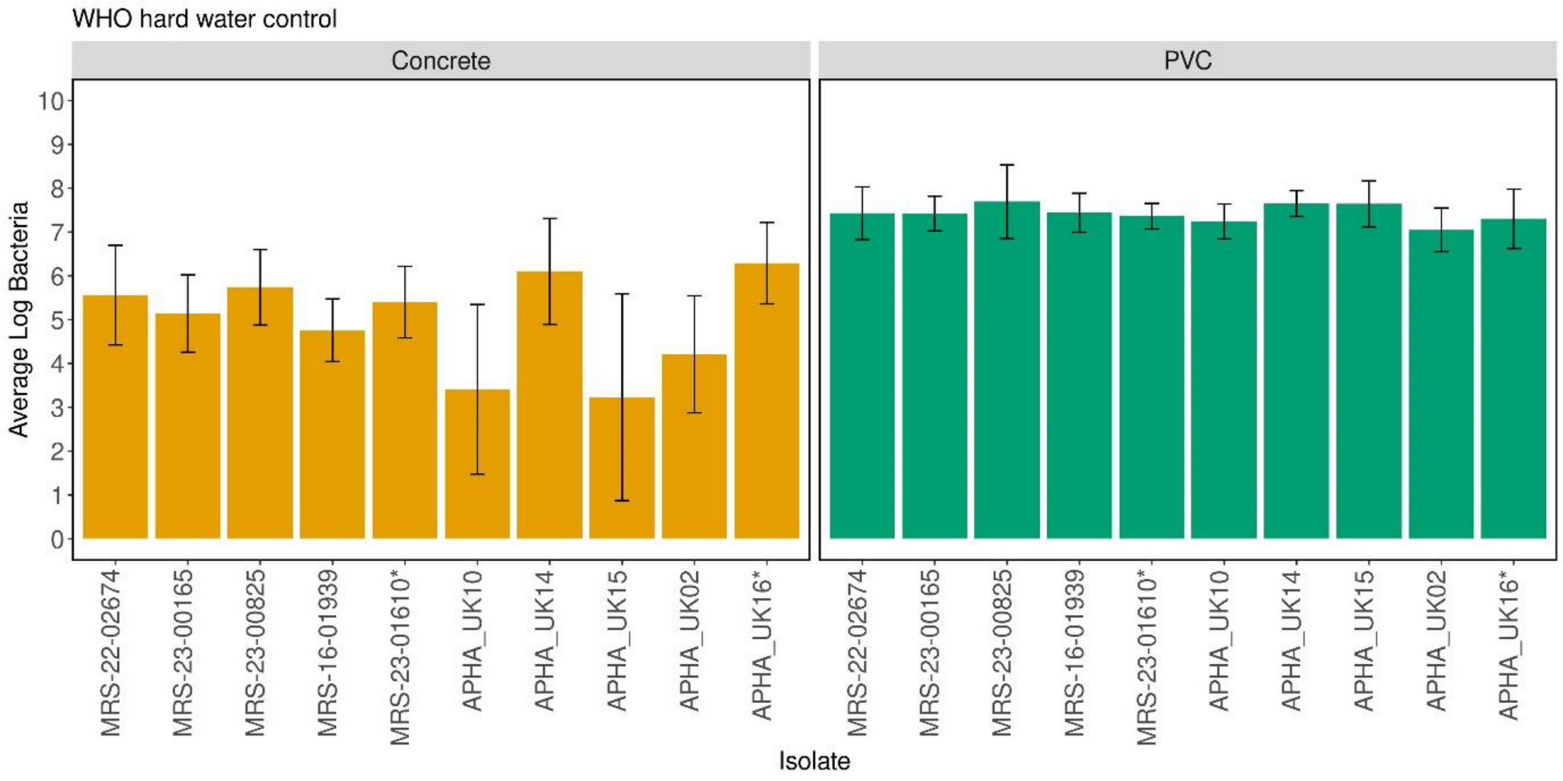
Bar graph depicting the average (*n*=10 replicates) log bacteria per coupon (Limit of detection=1.5) for the concrete surface (left, orange) and the PVC (right, green) when exposed to the WHO hard water. Error bars indicate variation between replicates. The asterisk (*) depicts isolates without pESI/pESI-like plasmids.

A starting inoculum of between 7.50×10^6^ and 2.38×10^9^ c.f.u. ml^−1^ was achieved. Disinfectant concentrations were selected with guidance from the MIC results. Biofilm formation (average log bacteria per coupon) was greater on PVC than concrete for all isolates (Fig. 8). The range in biofilm formation between all ten isolates was higher for the concrete than PVC (3.11 log bacteria per PVC coupon versus 5.41 log bacteria per concrete coupon). Within individual isolates, log bacteria per coupon varied up to 2.75 log on the PVC and up to 5.52 log on the concrete. A significant difference (*P≤*0.05) in the log bacteria was found between the two surfaces for five of the isolates (APHA_UK10, MRS-22-02674, MRS-23-00165, MRS-16-01939 and MRS-23-01610); biofilm formation was greater on PVC than concrete. A significant difference (*P*≤0.05) in biofilm formation was found between all ten isolates on concrete using the Kruskal–Wallis test; however, post hoc analysis (Dunn test) did not identify significant differences between strains, likely due to the small sample size. No significant difference in biofilm formation was found between the ten isolates on the PVC.

**Fig. 9.**
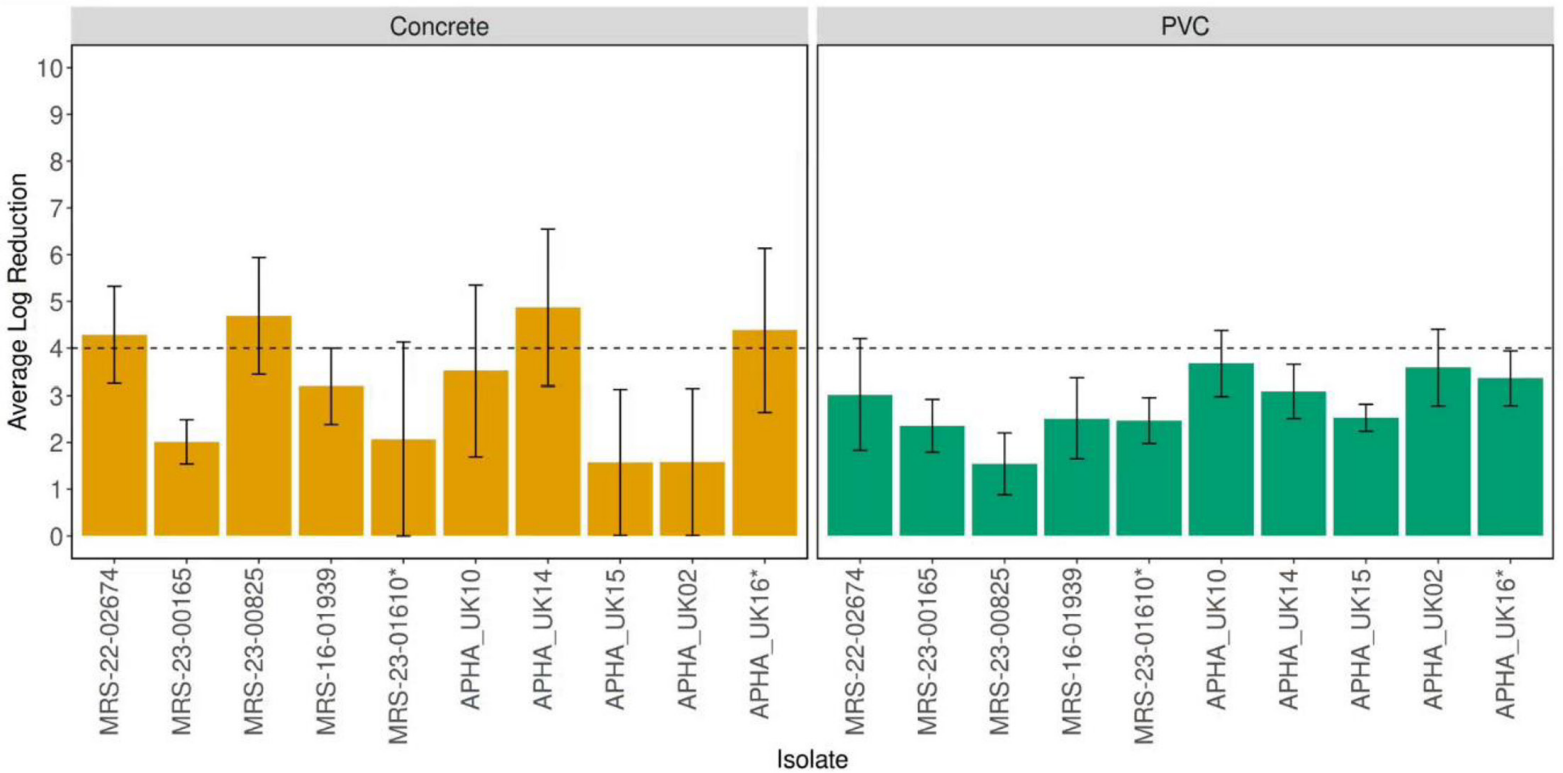
Bar graph depicting the average log reduction in bacteria per coupon (Limit of detection=1.5) after 30-min exposure to the aldehyde–QAC (1:528) product. Biofilm was grown on either PVC (green) or concrete (orange) coupons for 72 h at 20 °C. Error bars indicate sd between six replicates (three biological, two technical). The asterisk (*) depicts isolates without the pESI/pESI-like plasmid. The dotted line indicates 4 log reduction; ≥4 log reduction is deemed effective disinfection against biofilm.

Fig. 9 shows the average log reduction in biofilm following 30-min disinfection with the aldehyde–QAC product (1:528 concentration). The average log reduction on concrete ranged between 1.57 and 4.87 across all ten isolates; on PVC, it ranged between 1.55 and 3.68. Variation within individual isolates was greater for biofilm formed on concrete (up to 4.42 log) compared to PVC (up to 3.49 log). The disinfectant achieved a >4 average log reduction in four of the isolates on concrete, whereas no isolates achieved >4 average log reduction on PVC. No isolates achieved >4 log reduction across all three biological replicates. A significant difference (*P*≤0.05, paired t-test) in log reduction following disinfection was found between the two surfaces for two isolates (MRS-22-02674 and MRS-23-00825), with the disinfectant showing greater efficacy on concrete. A significant difference (*P*≤0.05, Kruskal–Wallis and ANOVA) in log reduction between the ten isolates was identified on both concrete and PVC surfaces, respectively, yet as previously described, post hoc tests failed to identify significance between strains.

**Fig. 10.**
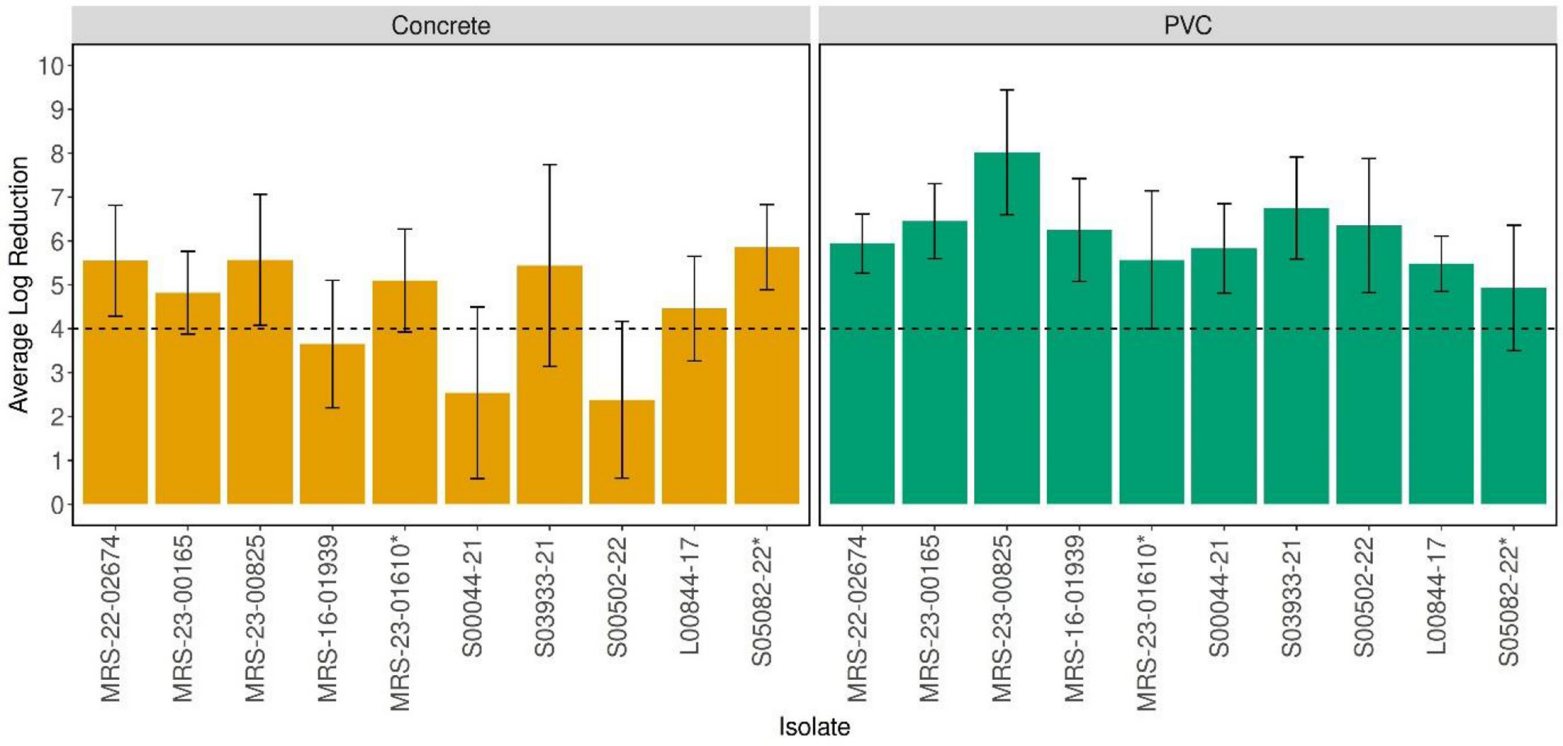
Bar graph depicting the average log reduction in bacteria per coupon (Limit of detection=1.5) after 30-min exposure to the chlorocresol 2 (1:150) product. Biofilm was grown on either PVC (green) or concrete (orange) coupons for 72 h at 20 °C. Error bars indicate sd between six replicates (three biological, two technical). The asterisk (*) depicts isolates without the pESI/pESI-like plasmid. The dotted line indicates 4 log reduction; ≥4 log reduction is deemed effective disinfection against biofilm.

Fig. 10 shows the average log reduction in biofilm following 30-min disinfection with the chlorocresol product (1:150 concentration). The average log reduction on concrete ranged between 2.38 and 5.86 and on PVC between 5.48 and 8.02. Variation within individual isolates was greater for biofilm formed on concrete (up to 6.68 log) compared to PVC (up to 4.67 log). The disinfectant achieved a >4 average log reduction in seven of the isolates on concrete (three strains achieved >4 log reduction across all three biological replicates) and for all isolates on PVC (two strains did not achieve >4 log reduction in all three biological replicates). However, it should be noted that more biofilm formed on the PVC than the concrete, thus allowing accurate calculation of log reduction. Due to the lower growth on the concrete, some isolates were found to have no growth following disinfection; due to this, an accurate log reduction is unable to be calculated. Therefore, the log reduction may be greater than detected for the concrete coupons. A significant difference (*P*≤0.05, paired t-test) in log reduction following disinfection was found between the two surfaces for only one isolate (APHA_UK15), with the disinfectant showing greater efficacy against the bacteria on the PVC surface. As seen in the QAC-aldehyde product, a significant difference (*P*≤0.05, Kruskal–Wallis and ANOVA) in log reduction between the ten isolates was identified on both concrete and PVC surfaces, respectively. However, again, post hoc tests (Dunn test) failed to identify the significance between strains.

**Fig. 11.**
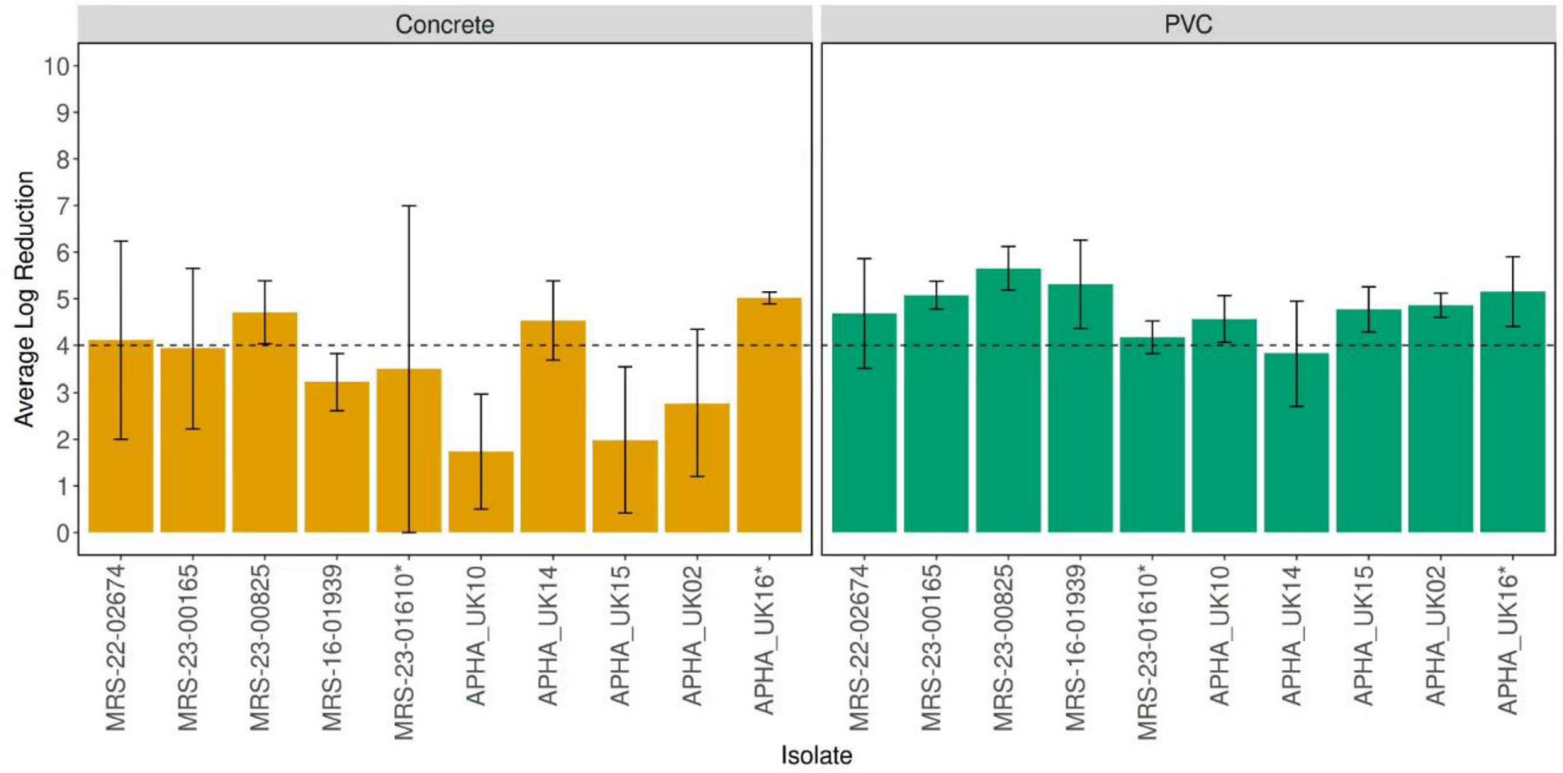
Bar graph depicting the average log reduction in bacteria per coupon (Limit of detection=1.5) after 30-min exposure to the peroxymonosulphate (1:160) product. Biofilm was grown on either PVC (green) or concrete (orange) coupons for 72 h at 20 °C. Error bars indicate sd between six replicates (three biological, two technical). The asterisk (*) depicts isolates without the pESI/pESI-like plasmid. The dotted line indicates 4 log reduction; ≥4 log reduction is deemed effective disinfection against biofilm.

Fig. 11 shows the average log reduction in biofilm following 30-min disinfection with the peroxymonosulphate product (1:160 concentration). The average log reduction on the concrete surface ranged between 1.74 and 5.02 across all ten isolates; on PVC, it ranged between 3.83 and 5.65. Variation within individual isolates was greater for biofilm formed on concrete (up to 5.03 log) compared to PVC (up to 1.91 log). The disinfectant achieved a >4 average log reduction in four of the isolates on concrete (only one isolate achieved a >4 log reduction in three biological replicates) and for nine of the isolates on PVC (two of which did not achieve >4 log reduction in all three biological replicates). However, as previously discussed, the log bacterial biofilm grown on the surfaces should be considered for its limitations when calculating log reduction. A significant difference (*P*≤0.05, paired t-test) in log reduction following disinfection was found between the two surfaces for five isolates (APHA_UK10, APHA_UK15, APHA_UK02, MRS-23-00825 and MRS-23-01610), with the disinfectant showing greater efficacy against the bacteria on PVC. Again, a significant difference (*P*≤0.05, Kruskal–Wallis) in log reduction between the ten isolates was identified on the concrete surface yet not following post hoc tests (Dunn test). No significant difference (*P*≤0.05, Kruskal–Wallis) was identified between the ten isolates on PVC.

## Discussion

The rapid increase in prevalence and persistence of the *Salmonella* serovar Infantis in recent decades has been attributed to the mega-plasmid, pESI. The plasmid has been identified globally, with high mosaicism, and found to harbour multiple resistances to antibiotics classified as high-priority and critically important for use in human and animal medicine [8–10, 12–14, 19–28]. The persistence and dissemination of the plasmid is of increasing concern to public health, and the mechanisms involved are poorly understood. The current study aimed to compare disinfectant efficacy and biofilm formation of pESI and pESI-like plasmids isolated in Austria and the UK.

Eight isolates which harboured pESI or pESI-like plasmids were selected for screening. Only short-read sequencing was available at the time of the study; thus, comparison of strains is limited by the constraints of short-read technology. For example, following Illumina sequencing, the plasmids were not circularized, whereas this would have been possible if long-read technology was employed. In this study, to obtain plasmid sequences, mob_recon was used to reconstruct the plasmids from short-read assemblies. However, the accuracy of reconstruction may be limited. Seven plasmids were reconstructed to ~280 kb – typical of pESI plasmids – whereas MRS-16-01939 was reconstructed to ~170 kb (Material S3). MRS-16-01939 harboured no antibiotic resistance genes on the plasmid, only chromosomally located fluoroquinolone resistance, as previously seen [1]; this strain potentially harboured a truncated version of the pESI plasmid due to a previous evolutionary event whereby part of the plasmid was lost or only a partial sequence of the plasmid was obtained.

The eight isolates were found to cluster depending on source of isolation (UK or Austria), suggesting most of the isolates to be epidemiologically distinct (<5 SNPs is considered a measure of high epidemiological link [51]). The Austrian isolates were more genomically similar than the UK isolates. MRS-22-02674 and MRS-23-0165 were most closely linked, isolated a year apart (2022 and 2023, respectively) and sourced from the same poultry farm. Likewise, the UK isolates APHA_UK15 and APHA_UK14 were sourced from the same poultry farm, again 1 year apart. However, APHA_UK15 harbours a beta-lactam resistance gene (*bla*_CTX-M-1_) on its pESI-like plasmid, whereas APHA_UK14 does not, suggesting a genetic alteration event has occurred whereby the *bla*_CTX-M-1_ gene has either been lost or gained. Furthermore, other studies have identified a link in isolates which harbour beta-lactam resistance genes and their trimethoprim resistance gene; those harbouring the *bla*_CTX-M-1_ gene are found to harbour the dfrA1 gene (as found here), whereas those harbouring the *bla*_CTX-M-65_ gene harbour the *dfrA14* gene [56]. Two of the Austrian isolates harboured an additional incompatibility replicon (IncX1); pESI isolates harbouring this additional incompatibility replicon were also found in the USA [14].

All ten isolates were evaluated for biofilm formation in the standardized CVA. Variation in biofilm formation was observed. This variation seemed strain-dependent and ranged with incubation duration. Biofilm formation could not be linked to the presence of the pESI plasmid when assessed following 72- and 120-h incubation. These results align with Bezek *et al*. [57]; however, they found that increasing incubation up to 168 h did suggest a relationship between pESI and biofilm formation. As found following core genome analysis, there was marginally more variation between the five UK isolates and the five Austrian isolates when comparing biofilm formation. Nonetheless, the variation observed in biofilm formation between the isolates, as supported by other studies [58], suggests that biofilm formation is strain-dependent, as opposed to being influenced by the presence of the pESI plasmid.

Likewise, tolerance to disinfectant also seemed to be strain-dependent in the MIC/MBC method, as previously found [59]. Variation between the isolates appeared random and varied depending on the disinfectant product. Unlike the CVA, the UK isolates did not appear more variable in their disinfectant tolerance compared to the Austrian isolates, nor vice versa. MRS-22-02674 was the most variable isolate in comparison to the other isolates; however, this is unlikely due to the presence of the pESI plasmid, as it harbours an identical AMR profile and differs by only one core genome SNP from MRS-23-00165. Tolerance to antimicrobials can vary between strains because of only one SNP difference. As seen in this study, different SNPs in the *gyrA* gene confer resistance to nalidixic acid. Further studies identify measurable changes in strain tolerance following only one SNP mutation; for example, an SNP in the *fusA1* gene results in greater aminoglycoside resistance, virulence and biofilm formation in *Pseudomonas aeruginosa* [54].

Therefore, we could potentially have an SNP in MRS-22-02674 which results in a measurable change/variation in our findings compared to our other strains, but we would need to do further investigation to identify the SNP/confirm this.

Biofilm was also grown and assessed for disinfectant tolerance on surfaces. These surfaces included a porous (concrete) and non-porous (PVC) surface. Previous studies have found that the strength of biofilm formation on porous and non-porous surfaces varies depending on bacterial species (APHA unpublished). This study found higher biofilm formation for all ten *Salmonella* isolates on the PVC surface, compared to the concrete. Tuominen *et al*. [60] also found biofilm formation to be poorest on concrete compared to PVC and stainless steel in *Staphylococcus aureus*. Difficulty in removing the biofilm from the concrete coupons may contribute to this result (i.e. pores in the concrete may trap biofilm). The variation between and within the isolates also varied much more on the concrete than on the PVC. This may be a result of variation in the surface of the concrete coupons themselves. Studies have identified surface characteristics to be involved in biofilm formation [60–62], such as surface roughness, hydrophobicity and topography [63]. The coupons are made in-house in batches; thus, the surface may vary widely. Likewise, the variation between isolates may also be a result of the concrete surface, as opposed to strain-dependent variation. This is supported by the consistency between isolates observed on the PVC surface.

The lower and more variable biofilm formation on the concrete surface limits interpretation of disinfectant tolerance in this study. Some isolates were unable to be recovered on the concrete surface (in fewer cases on the PVC) following exposure to disinfectant. Consequently, the log reduction was unable to be accurately calculated. As a result, when compared to the PVC surface, which typically had a higher starting biofilm count, the efficacy of the disinfectant against biofilm on the concrete surface seemed poorer (lower log reduction). This limitation was a particular concern for the chlorocresol and peroxymonosulphate products, but less so for the aldehyde/QAC product.

Overall, the isolates were more tolerant (lower log reduction) to the aldehyde–QAC product when grown on the PVC surface than on concrete. Again, higher variation in tolerance was observed between the ten isolates on the concrete compared with the PVC. No significant difference was found in tolerance to the aldehyde–QAC product between the ten isolates on either surface. This suggests that any variation is strain dependent.

When exposed to both the chlorocresol and peroxymonosulphate product, the isolates were overall more tolerant when biofilm was formed on the concrete surface. However, as previously described, the lower initial biofilm formation on the concrete surface limits the interpretation of these results. Again, no significant variation was found between the ten isolates on either surface, suggesting variation because of methodology or strain.

The peroxymonosulphate and chlorocresol products were tested at half and third strength concentration, respectively, than their recommended concentration. The aldehyde–QAC product was tested at 16-fold weaker concentration than recommended. A 4-log reduction is deemed effective disinfection against biofilm (EN 14349, EN 13697 and EN 16437). Whilst this was achieved in at least one replicate for 100% of isolates for the chlorocresol, 85% of isolates for the peroxymonosulphate and in 60% of isolates for the aldehyde–QAC, a 4-log reduction was not always achieved across all three biological replicates. Therefore, the tested concentrations do not consistently achieve the desired 4-log reduction. It could be assumed that increasing the concentration to the recommended concentration would effectively eradicate the biofilm, particularly when mechanical removal is applied. However, the persistence of *S.* Infantis in agricultural, food and veterinary premises suggests this is not the case. Alternatively, the serovar persistence may suggest the inappropriate application of disinfectants [such as the use of incorrect concentration, reduced contact time, presence of organic matter (not studied here) or lack of mechanical removal [64]]. Alternatively, the persistence of the serovar may be a result of other factors (i.e. pathogenesis in the host). Consequently, further study needs to be done to understand why this serovar, harbouring the pESI plasmid, is so persistent in the environment.

In conclusion, biofilm formation, assessed via the CVA and the surface coupon assay, does not seem to be influenced by the presence of the pESI or pESI-like plasmid. Furthermore, disinfectant tolerance, when assessed in both planktonic and biofilm standardised models and against biofilm on surface, also does not seem to be influenced by the presence of the pESI or pESI-like plasmid. Variation observed in biofilm formation and biofilm disinfectant tolerance is deemed to be strain-dependent, influenced by methodology (i.e. surface) or varies depending on disinfectant product. Furthermore, the surface on which biofilm is formed and to which disinfectant is applied should be carefully considered when establishing suitable disinfection regimes both for general use and in the event of an outbreak of disease on farms, food processing and veterinary facilities.

## Supplementary material

10.1099/acmi.0.001190.v3 Supplementary Material 1.

## Abbreviations

AMR: antimicrobial-resistant
APHA: Animal and Plant Health Agency
ARG: antimicrobial resistant gene
CVA: crystal violet microtiter plate assay
EU: European Union
LB: Luria–Bertani
MBC: minimum bactericidal concentration
MDR: multi-drug resistant
pESI: plasmid of emerging *Salmonella* Infantis
PVC: polyvinyl chloride
QAC: quaternary ammonium compound
SBA: sheep blood agar
WGS: whole-genome sequencing
WHO: World Health Organization
