# Supplementary material for: Influence of the Salmonella Infantis pESI plasmid on disinfectant efficacy when in biofilm
Source: Access Microbiol. 2026 Jul 27;8(7):001190.v3. doi: 10.1099/acmi.0.001190.v3 (PMC13404209; doi:10.1099/acmi.0.001190.v3)
Supplement: Supplementary Material 1. [file acmi-8-01190-s001.pdf]

Supplementary material 1 - S. Infantis isolated in the United Kingdom (UK) from poultry broiler between 2011 and 2022. Following whole genome sequencing analysis of antibiotic resistance genes (starAMR and AMRFinderPlus) the isolates were grouped into antimicrobial resistance (AMR) profiles. Ten profiles were identified. The four isolates in bold were selected for wet laboratory testing based on the presence of resistance genes relevant to human and animal health, and similarity or difference to globally identified pESI and pESI-like plasmids.

AMR Profile	Isolate ID	Country	Host	Year	MLST	*ant(3'')-Ia*	*aph(3')-Ia*	*bla*$_{CTX-M-1}$	*bla*$_{TEM-1B}$	*dfrA1*	*dfrA14*	*sul1*	*lnu(G)*	*tet(A)*	*qacEΔ1*	*gyrA* (D87G)	*gyrA* (D87Y)	*gyrA* (S83Y)	*parC* (T57S)	IncFIB(pN55391)	IncFII(29)	IncX4
1	APHA_UK01	England/Wales	Broiler	2014	ST32	+	-	-	-	-	+	+	-	+	+	-	+	-	+	+	-	-
	**APHA_UK02**	**England/Wales**	**Broiler**	**2017**	**ST32**	**+**	**-**	**-**	**-**	**-**	**+**	**+**	**-**	**+**	**+**	**-**	**+**	**-**	**+**	**+**	**-**	**-**
	APHA_UK03	England/Wales	Broiler	2016	ST32	+	-	-	-	-	+	+	-	+	+	-	+	-	+	+	-	-
	APHA_UK04	England/Wales	Broiler	2013	ST32	+	-	-	-	-	+	+	-	+	+	-	+	-	+	+	-	-
2	APHA_UK05	England/Wales	Broiler	2014	ST32	-	-	-	-	-	-	-	-	-	-	-	-	+	+	+	-	-
3	APHA_UK06	England/Wales	Broiler	2015	ST32	+	-	-	-	-	-	+	-	+	+	+	-	-	+	+	+	-
4	APHA_UK07	England/Wales	Broiler	2015	ST32	+	-	-	-	-	-	+	-	+	+	+	-	-	+	+	-	-
5	APHA_UK08	England/Wales	Broiler	2011	ST32	+	-	-	-	-	-	+	-	+	+	-	-	+	+	+	-	-
6	APHA_UK09	England/Wales	Broiler	2015	ST32	+	-	-	+	-	-	+	+	+	+	+	-	-	+	+	-	+
7	**APHA_UK10**	**England/Wales**	**Broiler**	**2021**	**ST32**	**+**	**+**	**-**	**-**	**-**	**+**	**+**	**-**	**+**	**+**	**+**	**-**	**-**	**+**	**+**	**-**	**-**
	APHA_UK11	England/Wales	Broiler	2021	ST32	+	+	-	-	-	+	+	-	+	+	+	-	-	+	+	-	-
8	APHA_UK12	England/Wales	Broiler	2021	ST32	-	-	+	-	+	-	+	-	+	+	+	-	-	+	+	-	-
	APHA_UK13	England/Wales	Broiler	2022	ST32	-	-	+	-	+	-	+	-	+	+	+	-	-	+	+	-	-
9	**APHA_UK14**	**England/Wales**	**Broiler**	**2021**	**ST32**	**-**	**-**	**-**	**-**	**+**	**-**	**+**	**-**	**+**	**+**	**+**	**-**	**-**	**+**	**+**	**-**	**-**
10	**APHA_UK15**	**England/Wales**	**Broiler**	**2022**	**ST32**	**-**	**-**	**+**	**-**	**+**	**-**	**+**	**-**	**+**	**+**	**+**	**-**	**-**	**+**	**+**	**-**	**-**

Supplementary material 2 - A list of published pESI/pESI-like reference isolates utilised in phylogenetic analyses. The original isolate ID, country of origin, source, year of isolation, GenBank whole genome Accession Number, Publication reference and plasmid-located antimicrobial resistance genes are noted.

**Isolate ID**	**Country of Origin**	**Source**	**Year of isolation**	**GenBank Accession Number**	**Reference**	**Plasmid-located antimicrobial genes**
pESI_119944	Israel	Human	2008	ASRF00000000	(10)	*tetAR, sul1, dfrA14, aadA1,* and *qacEΔ1*
13017779/5	Italy	Chicken (Broiler)	2013	DAAREW000000000.1	(11)	*tetAR, sul1, dfrA1, dfrA14, qacEΔ1, aph(3')-Ic* and $bla_{CTX\text{-}M\text{-}1}$
13002124/1	Italy	Human	2013	DAATVX000000000.1		*tetAR, sul1, dfrA1, dfrA14, qacEΔ1, aph(3')-Ic* and $bla_{CTX\text{-}M\text{-}1}$
12037823/11	Italy	Chicken (Broiler)	2012	DAASFK000000000.1		*tetAR, sul1, dfrA1, dfrA14, qacEΔ1, aph(3')-Ic* and $bla_{CTX\text{-}M\text{-}1}$
13002124/34	Italy	Chicken (Broiler)	2013	DAATCB000000000.1		*tetAR, sul1, dfrA1, dfrA14, qacEΔ1, aadA1* and $bla_{CTX\text{-}M\text{-}1}$
2014AM-3028	United States of America	Human	2014	CP016412.1	(13)	*aph(4)-Ia, aac(3)-IVa, aadA1,* $bla_{CTX\text{-}M\text{-}65}$, *sul1, tetA,* and *dfrA14*
N55391	United States of America	Chicken (Broiler)	2014	CP016410.1		*aph(4)-Ia, aph(3)-Ic, aac(3)-IVa,* $bla_{CTX\text{-}M\text{-}65}$, *sul1, tetA,* and *dfrA14*
FSIS1502169	United States of America	Chicken (Broiler)	2015	CP016406.1		*aph(4)-Ia, aph(3’)-Ic, aac(3)-IVa, aadA1,* $bla_{CTX\text{-}M\text{-}65}$, *sul1, tetA,* and *dfrA14*

FSIS1502916	United States of America	Chicken (Broiler)	2015	CP016408.1		*aph(4)-Ia, aph(3')-Ic, aac(3)-IVa, aadA1, bla$_{CTX-M-65}$, sul1, tetA,* and *dfrA14*
114061	United Kingdom	Human	2015	CP070302.1	(12)	*tetAR, sul1, dfrA14, aac(3)-IVa, aph(3')-lc, aph(4)-la, qacEΔ1 and bla*$_{CTX-M-65}$
144-13	Switzerland	Food	2013	NAOX00000000.1	(18)	*tetAR, sul1, dfrA14, bla*$_{CTX-M-65}$, and *qacEΔ1*
423_13	Brazil	Human	2013	CP093373	(28)	*aph(4)-Ia, aac(6')-Iaa, aac(3)IV, aadA1, sul1, tetA, dfrA14, bla$_{CTX-M-65}$*
VGNKI-11	Russia	Chicken (Broiler)	2017	VSBW01000023.1	(29)	*tetAR, aadA1, aac6-ly, dfrA14, sul1*

Supplementary material 3 – Whole genome sequencing quality metrics. QUAST (Quality Assessment Tool) (49) was used to evaluate the quality of the Illumina short-read assemblies (acceptable parameters: N50 >50,000, contigs <200 and BPs between 4.5 million and 5.2 million) and to assess reconstruction of plasmids following use of mob_recon.

	MRS-23-00825	APHA_UK10	APHA_UK15	APHA_UK14	APHA_UK02	MRS-22-02674	MRS-23-00165	MRS-16-01939	APHA_UK16	MRS-23-01610
Whole Genome										
**# contigs**	59	99	45	50	77	112	125	146	39	55
**Largest contig**	1194092	319793	1195370	850294	1028713	553764	317904	332511	1194834	1099708
**Total length**	4981303	4876427	4920535	4918270	4922336	4953103	4905201	4756925	4670417	4577437
**GC (%)**	52.21	52.19	52.14	52.15	52.1	52.1	52.1	52.19	52.14	52.31
**N50**	201820	94152	227778	217634	183529	114303	77417	90175	263193	157269
**N90**	54779	30849	89702	83214	57217	30011	26805	29179	89545	68808
Plasmid										
**# contigs**	7	9	5	8	14	17	14	8		
**Largest contig**	130780	130649	130655	130655	76620	63593	65043	67360		
**Total length**	275362	282800	273707	273191	277823	273554	272297	170435*		
**GC (%)**	50.12	50	50.06	50.07	49.95	50.22	50.33	49.24		
**N50**	86993	104579	102114	83214	62660	40036	26877	32217		
**N90**	16509	24480	24461	18912	10128	6923	13075	24293		

*a truncated plasmid sequence was generated for MRS-16-01939

Supplementary material 4 – BioProject and Accession Number details for the S. Infantis isolated in the United Kingdom (UK) and Austria utilised in this study.

Strain ID	BioProject	Accession Number
MRS-22-02674	PRJNA1381483	SAMN54106534
MRS-23-00165		SAMN54106535
MRS-23-00825		SAMN54106536
MRS-23-01610		SAMN54106537
APHA_UK10	PRJNA1403979	SAMN54691484
APHA_UK11		SAMN54691485
APHA_UK12		SAMN54691486
APHA_UK13		SAMN54691487
APHA_UK14		SAMN54691488
APHA_UK15		SAMN54691489
